# Identification and validation of druggable targets for cataract using mendelian randomization: functional insights from multi-omics and an oxidative stress model

**DOI:** 10.3389/fmed.2026.1741371

**Published:** 2026-04-16

**Authors:** Min Lin, Jie Zeng

**Affiliations:** 1 Department of Ophthalmology, Fujian Provincial Geriatric Hospital, Fujian Provincial Hospital North Branch, Fuzhou, China; 2 Department of Ophthalmology, Fujian Medical University Affiliated Min Dong Hospital, Ningde, China

**Keywords:** cataract, drug repurposing, druggable targets, GSTM1, mendelian randomization, oxidative stress

## Abstract

**Objective:**

To identify druggable genes associated with cataract and investigate their functional roles under oxidative stress, thereby providing potential therapeutic targets.

**Methods:**

Multi-omics data were integrated, and expression quantitative trait loci (eQTL) and protein quantitative trait loci (pQTL) analyses, along with Mendelian randomization (MR), were performed to assess the causal relationships between gene/protein expression and cataract risk. Phenome-wide association studies (PheWAS), the creation of protein–protein interaction networks, drug prediction, and molecular docking were further performed to evaluate their functional relevance and potential for drug development. In addition, an oxidative stress model was established by treating SRA01/04 lens epithelial cells with H_2_O_2_, and cell viability, proliferation, apoptosis, and hub gene expression were assessed using CCK8, EdU, flow cytometry, and qPCR.

**Results:**

Among 2,532 drug-associated genes, 35 eQTL genes and 31 pQTL genes were identified, with DKK3, GSTM1, and KIR2DS4 showing significant associations in both analyses. PheWAS revealed no major adverse effects, and drug prediction and molecular docking suggested GSTM1 as the most promising target. *In vitro*, H_2_O_2_ suppressed SRA01/04 cell viability and proliferation while promoting apoptosis in a dose-dependent manner. qPCR results showed that oxidative stress upregulated DKK3 and downregulated GSTM1 expression, both in a dose-dependent manner, consistent with MR findings, supporting DKK3 as a risk factor and GSTM1 as a protective factor for cataract.

**Conclusion:**

This study identified and validated DKK3 and GSTM1 as key genes in cataract pathogenesis. By integrating genetic analyses with functional experiments, our findings provide new insights into the molecular mechanisms of cataract and establish a theoretical basis for drug development and repurposing.

## 1 Introduction

Cataract is one of the leading causes of blindness worldwide, primarily characterized by lens opacity, which impairs normal light transmission and results in progressive vision loss or even blindness (1). The pathogenesis of cataract is complex and influenced by both environmental factors—such as ultraviolet radiation and diabetes—and individual genetic background (2). With the global trend of population aging, the prevalence of cataract continues to rise. At present, cataracts impact over 95 million individuals, with more than half of those over 65 years old affected, posing a significant public health challenge that diminishes the quality of life for the elderly (3).

At present, cataract can be effectively treated only by surgery. However, some patients still experience postoperative complications, such as intraocular lens decentration or dislocation, ocular perforation caused by anesthesia, optic nerve injury leading to vision loss, and infectious endophthalmitis (4). Although various drugs, including antioxidants and aldose reductase inhibitors, have been developed in recent years to delay the onset and progression of cataract, no drug has yet been proven effective in large-scale clinical practice (5, 6). Therefore, exploring the molecular mechanisms underlying cataract and identifying novel therapeutic targets is both urgent and necessary.

With the rapid development of computational biology and systems pharmacology, in silico approaches have increasingly been used to facilitate drug discovery and target identification (7). In particular, computational frameworks for drug–target interaction (DTI) prediction allow the integration of heterogeneous biological and chemical information, providing new opportunities to prioritize candidate therapeutic targets and explore drug repurposing strategies. For example, Zhou et al. developed computational models that combine molecular descriptors with biological features to improve the prediction of drug–target interactions (8). More recently, Qian et al. reported that advanced feature representation and deep learning–based models can further enhance the performance of DTI prediction and accelerate drug discovery processes (9). These advances highlight the growing importance of integrating computational pharmacology with multi-omics data to better understand disease mechanisms and identify potential therapeutic targets.

In recent years, the rapid advancement of genomics has brought significant breakthroughs in ophthalmic disease research. In particular, expression quantitative trait loci (eQTL) and protein quantitative trait loci (pQTL) analyses have emerged as powerful tools for identifying disease-associated genetic variants (10, 11). By mapping regulatory variants that influence gene or protein expression levels, eQTL and pQTL analyses help to elucidate the functional mechanisms of cataract-associated loci identified through genome-wide association studies (GWAS), thereby facilitating the discovery of potential therapeutic targets (12, 13). Simultaneously, the notion of the druggable genome has boosted the practical significance of these results, underlining the potential for therapeutic targeting of these genes. Identifying genetically targetable loci provides a clear direction for creating new therapeutic approaches for cataracts (12, 14).

Building upon this, Mendelian randomization (MR), a causal inference method based on genetic variation, can effectively overcome confounding and reverse causation issues commonly seen in observational studies. In recent years, MR has been widely applied to explore causal relationships between eQTLs/pQTLs and phenotypes and has demonstrated great promise in drug target validation and druggable gene prioritization (15, 16). Several studies have already identified candidate genes and functional variants associated with cataract using this approach (12). However, many genes identified by genome-wide MR analyses lack clear pharmacological tractability. Therefore, focusing MR analyses on the druggable genome may facilitate the prioritization of targets with greater therapeutic potential. This study integrates multi-omics data, combining druggable genomic eQTL and pQTL data, and applies MR to explore potential drug targets for cataracts. Additionally, an *in vitro* experiment using H_2_O_2_-induced lens epithelial cells is conducted to validate the effects on cell proliferation, apoptosis, and the expression of key genes. This aims to address current gaps in the research and contribute to the advancement of precision treatments for cataracts.

## 2 Materials and methods

**Figure 1:**
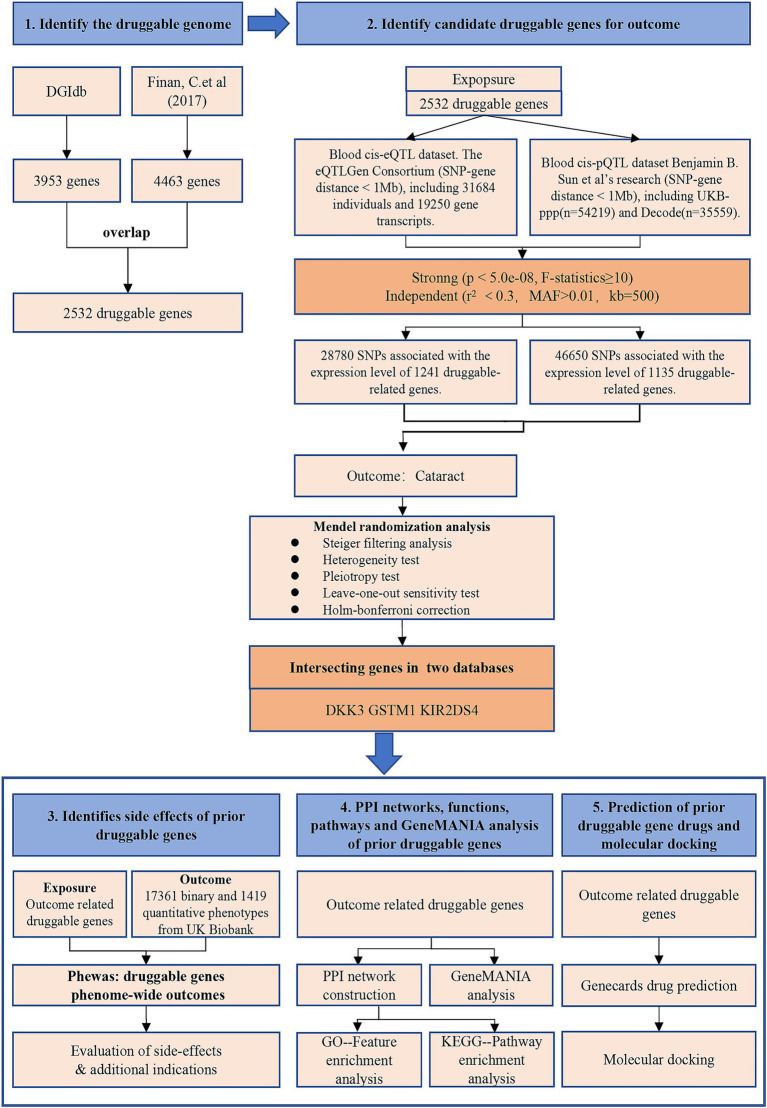
Overview of this study.

An illustration of the study’s overall design is shown in Figure 1, with further details on materials and methods provided below.

### 2.1 Data sources

The druggable genes used in this study were obtained from the Drug–Gene Interaction Database (DGIdb v4.2.0)^1^ (17) and the druggable genome framework proposed by Finan et al. (18). DGIdb is a widely used integrative resource that aggregates drug–gene interaction information from multiple curated databases and literature sources, enabling systematic identification of genes with known or potential pharmacological relevance. In contrast, the framework proposed by Finan et al. provides a genetically informed classification of the druggable genome, prioritizing genes with evidence supporting their suitability as therapeutic targets. By combining these two complementary resources, we aimed to obtain a comprehensive and genetically supported set of druggable genes for downstream analyses.

We employed eQTL and pQTL data obtained from human blood samples (Table 1). Blood-derived molecular QTL datasets are widely used in Mendelian randomization studies because their large sample sizes provide strong statistical power and reliable genetic instruments for causal inference and drug target prioritization. The eQTLGen Consortium^2^ provided cis-eQTL data, encompassing transcript-level details for 19,250 genes from 31,684 individuals. Blood-based cis-pQTL data were sourced from two proteomic studies: the UK Biobank Pharma Proteomics Project (UKB-PPP),^3^ which encompasses data from 54,219 individuals and a genome-wide association study led by Egil Ferkingstad et al. (19) which analyzed plasma protein levels using 4,907 aptamers in 35,559 Icelandic individuals (Table 1).

**Table 1:** Brief information of the GWAS database in MR Research.

Type	Data source	Sample size	Cases	Population
eQTLs	eQTLGen	31,684	–	European
pQTLs	UKB-PPP	54,219	–	European
pQTLs	Decode	35,559	–	European
Cataract	GWAS catalog (GCST90043785)	456,348	14,867	European

Genetic association data for cataract were obtained from the GWAS Catalog. Summary-level statistics were based on 456,348 individuals of European ancestry, including 14,867 cases and 441,481 controls (Table 1).

### 2.2 Ethical statement

Informed consent from participants was obtained in all the original studies. Thus, no further ethics approval was needed for this aspect of our study.

### 2.3 Instrumental variable selection

Eligible instrumental variables (IVs) must meet three core assumptions: (1) the IV is directly associated with the exposure; (2) the IV is independent of any confounders; and (3) the IV is not associated with the outcome. First, potential druggable genes were cross-referenced with human blood-based eQTL and pQTL datasets to obtain corresponding variant datasets for druggable genes (Supplementary Figure S1), which illustrates the overall workflow for instrumental variable selection and MR analysis. SNPs were extracted from the eQTL and pQTL datasets using a genome-wide significance threshold of *p* < 5 × 10^−8^. To ensure independence among instrumental variables, linkage disequilibrium (LD) pruning was performed with an *r*^2^ threshold of < 0.3 within a 500 kb window. Variants with minor allele frequency (MAF) < 0.01 were excluded. Palindromic SNPs and those directly associated with the outcome (*p* < 5 × 10^−8^) were also removed. To minimize weak instrument bias, the strength of each instrumental variable was evaluated using the F statistic, calculated based on the proportion of variance explained by the SNP. SNPs with F statistics greater than 10 were retained as valid instrumental variables according to commonly accepted criteria in Mendelian randomization studies. The distribution of F statistics for the included SNPs is provided in the Supplementary Tables. Outlier SNPs were further detected and removed using the MR-PRESSO method.

### 2.4 Mendelian randomization and Steiger filtering

Following the identification of the IVs, five MR approaches were employed to determine the associations between exposure and outcome: The techniques used are MR-Egger regression, inverse-variance weighted method, weighted median estimator, weighted mode, and simple mode. The IVW method was used to assess causal relationships, and the effect of individual SNPs was calculated using the Wald ratio. Holm–Bonferroni correction was applied for multiple testing. Directional horizontal pleiotropy was evaluated using the MR-Egger intercept test. A non-significant MR-Egger intercept (*p* > 0.05) was considered to indicate no evidence of directional pleiotropy. Sensitivity of the MR estimates was further assessed using leave-one-out analysis. Heterogeneity among SNPs was evaluated using Cochran’s Q statistic and the I^2^ statistic. Finally, the direction of causality was assessed using the Steiger filtering approach. The analyses were carried out with the TwoSample MR package in R version 4.1.0. The version of the TwoSampleMR package used in this study was 0.5.6, which is available at https://github.com/MRCIEU/TwoSampleMR.

### 2.5 Phenome-wide association study (PheWAS)

A gene-level PheWAS was carried out using data from the AstraZeneca PheWAS portal^4^ to examine whether druggable genes might impact other traits (20), featuring 17,361 binary traits along with 1,419 quantitative traits (21). Associations with *p* < 5 × 10^−8^ were considered statistically significant.

### 2.6 PPI network, functional and pathway enrichment analysis, and GeneMANIA

To study the interactions between MR-prioritized druggable genes and delve deeper into their connection to cataract development, these genes were entered into the STRING database^5^ with a confidence score of at least 0.7 (22). Cytoscape was used to visualize the resulting protein–protein interaction (PPI) network.

The clusterProfiler R package facilitated the enrichment analyses for Gene Ontology (GO) and Kyoto Encyclopedia of Genes and Genomes (KEGG) pathways, applying a significance level of *p* < 0.05. To gain more insight into the functional and co-expression aspects of druggable genes, GeneMANIA was employed to develop a gene co-expression network.

### 2.7 Drug prediction and molecular docking

Potential pharmaceuticals targeting the identified druggable genes were extracted from the GeneCards database^6^ (23), and the drug–gene interaction pairs were imported into Cytoscape for visualization of the candidate gene–drug interaction network.

Using CB-Dock2, molecular docking was performed to determine the binding affinities between drugs and their target genes.^7^ The initial step involved obtaining drug structures from the PubChem database. The next step involved retrieving target proteins from UniProt and the Protein Data Bank to obtain high-resolution receptor structures. Water molecules were removed using AutoDock, and molecular docking and visualization were performed with CB-Dock2. Compounds with a Vina score > − 5 were excluded (24, 25), allowing for rapid identification of molecules with potential drug activity.

### 2.8 Construction of the H_2_O_2_-induced oxidative stress model

SRA01/04 lens epithelial cells were cultured in DMEM medium containing 10% fetal bovine serum (FBS) and 1% penicillin/streptomycin, in a 37 °C, 5% CO_2_ incubator. The cells were passaged when they reached 80–90% confluence. Cells were treated with various concentrations of H_2_O_2_ (100–1,000 μM) for 24 or 48 h to establish the oxidative stress model. The control group was treated with an equal volume of PBS.

### 2.9 CCK8 assay for cell viability

Cell viability was assessed using the CCK8 assay kit (Beyotime, C0038). At the end of the experiment, 10 μL of CCK8 working solution was added to each well, and cells were incubated at 37 °C for 2 h. Optical delnsity (OD) values were measured at 450 nm using a microplate reader. Cell viability was calculated by the ratio of OD values between the treated group and control group, and a cell viability curve was generated to reflect the impact of H_2_O_2_ on cell growth.

### 2.10 EdU assay for cell proliferation

Cell proliferation was assessed using the EdU assay kit (Sangon Biotech, E607204). Cells were first treated with H_2_O_2_ for 24 h to induce oxidative stress. The H_2_O_2_ concentrations used in this study were selected based on preliminary CCK8 screening experiments and previously reported oxidative stress models in lens epithelial cells. Cells were then incubated with 10 μM EdU at 37 °C for 2 h. After discarding the medium, cells were fixed with 4% paraformaldehyde for 30 min, permeabilized with 0.5% Triton X-100 for 10 min, and then the reaction system was prepared as per the instructions. TAMRA red fluorescence dye was added for a 30-min reaction. Finally, cells were stained with Hoechst 33342, and proliferation was observed and photographed under a fluorescence microscope. The EdU positive rate was calculated to evaluate the level of cell proliferation.

### 2.11 Flow cytometry for apoptosis detection

Detection of cell apoptosis was carried out using the Annexin V-FITC/PI double staining method (LianKe Biological, China, AP101). After treatment, cells were collected, washed with PBS, and resuspended in binding buffer. Annexin V-FITC and PI were added and incubated at room temperature in the dark for 5 min. Unstained controls and single-stained controls were used to establish gating and fluorescence compensation. Apoptotic populations were identified using a standard Annexin V/PI quadrant gating strategy: Annexin V^−^/PI^−^ cells were considered viable cells, Annexin V^+^/PI^−^ cells were classified as early apoptotic cells, Annexin V^+^/PI^+^ cells as late apoptotic cells, and Annexin V^−^/PI^+^ cells as necrotic cells. Fluorescent signals were detected using flow cytometry, and data were analyzed using FlowJo software.

### 2.12 qPCR for hub gene expression

Total RNA from each group of cells was extracted using the SteadyPure Rapid RNA Extraction Kit (Akoray, China, AG21023), and RNA purity and concentration were determined by NanoDrop. Reverse transcription and qPCR were performed using the PrimeScript™ RT reagent Kit (TaKaRa, RR047A). Primers were synthesized by Sangon, and the sequences are shown in Supplementary Table S9. GAPDH was used as the internal reference gene. Relative mRNA expression levels of target genes were normalized to GAPDH and calculated using the 2^−ΔΔCt^ method.

### 2.13 Statistical analysis

Data are presented as mean ± standard deviation (mean ± SD). Inter-group comparisons were performed using one-way analysis of variance (one-way ANOVA). GraphPad Prism 9.0 software was used for data analysis, and a *p*-value of less than 0.05 was deemed statistically significant.

## 3 Results

### 3.1 Candidate druggable genes

We downloaded 3,953 potential drug target genes from DGIdb v4.2.0 and extracted 4,463 druggable genes from the study by Finan C et al. To ensure that the selected druggable genes were both reliable and more likely to represent effective drug targets, we integrated the datasets and obtained 2,532 unique druggable genes—named according to the HUGO Gene Nomenclature Committee (HGNC)—for downstream analysis.

### 3.2 Mendelian randomization analysis

After filtering, 28,780 SNPs associated with the transcript expression of 1,241 druggable genes were identified from the cis-eQTL dataset (Supplementary Table S1). From two proteomic cis-pQTL datasets, 46,650 SNPs associated with the protein expression of 1,135 druggable genes (corresponding to 803 unique proteins) were selected (Supplementary Table S2).

Using these instrumental variables, we systematically evaluated the causal effects of druggable genes on cataract. The eQTL-based MR analysis identified 320 druggable genes linked to cataract phenotypes (*p* < 0.05). In the pQTL-based MR analysis, 283 druggable gene products (245 unique proteins) were associated with cataract phenotypes (*p* < 0.05).

After Holm–Bonferroni correction, 42 druggable genes remained significantly associated with cataract in the eQTL dataset (*p* < 0.05; Supplementary Table S3), and 38 druggable gene products (35 unique proteins) remained significant in the pQTL dataset (*p* < 0.05; Supplementary Table S4).

**Figure 2:**
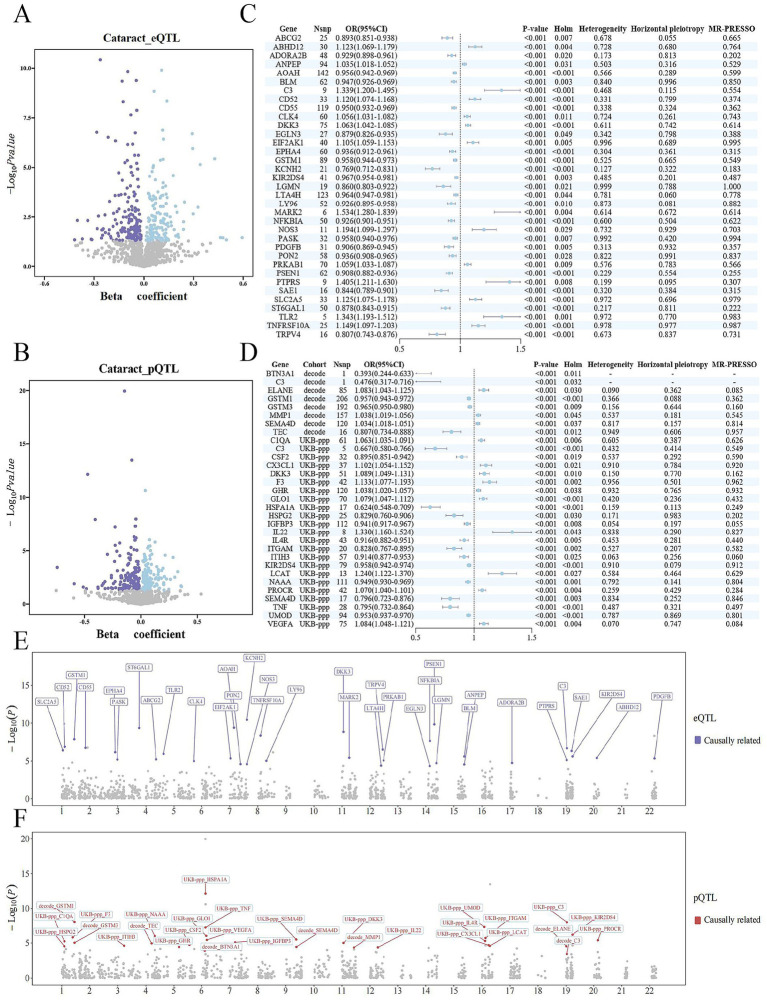
Mendelian randomization results of druggable genes associated with cataract. **(A)** Volcano plot showing the MR results of druggable gene expression (eQTL) and cataract; **(B)** Volcano plot showing the MR results of druggable protein expression (pQTL) and cataract; **(C)** Forest plot of selected druggable genes based on eQTL MR analysis; **(D)** Forest plot of selected druggable genes based on pQTL MR analysis; **(E)** Manhattan plot of MR results for eQTL–cataract associations; **(F)** Manhattan plot of MR results for pQTL–cataract associations; Significance threshold: *p* < 0.05.

After removing results with heterogeneity or horizontal pleiotropy, 35 druggable genes in the eQTL dataset were identified as significantly associated with cataract (*p* < 0.05; Figures 2A, C, E; Supplementary Table S5), along with 31 druggable gene products (29 unique proteins) in the pQTL dataset (*p* < 0.05; Figures 2B, D, F; Supplementary Table S6). Sensitivity analyses using leave-one-out tests showed that the causal estimates were not driven by any single SNP (Supplementary Figures S2 and S3), indicating that the MR results were robust.

### 3.3 Integration of multi-omics evidence

**Figure 3:**
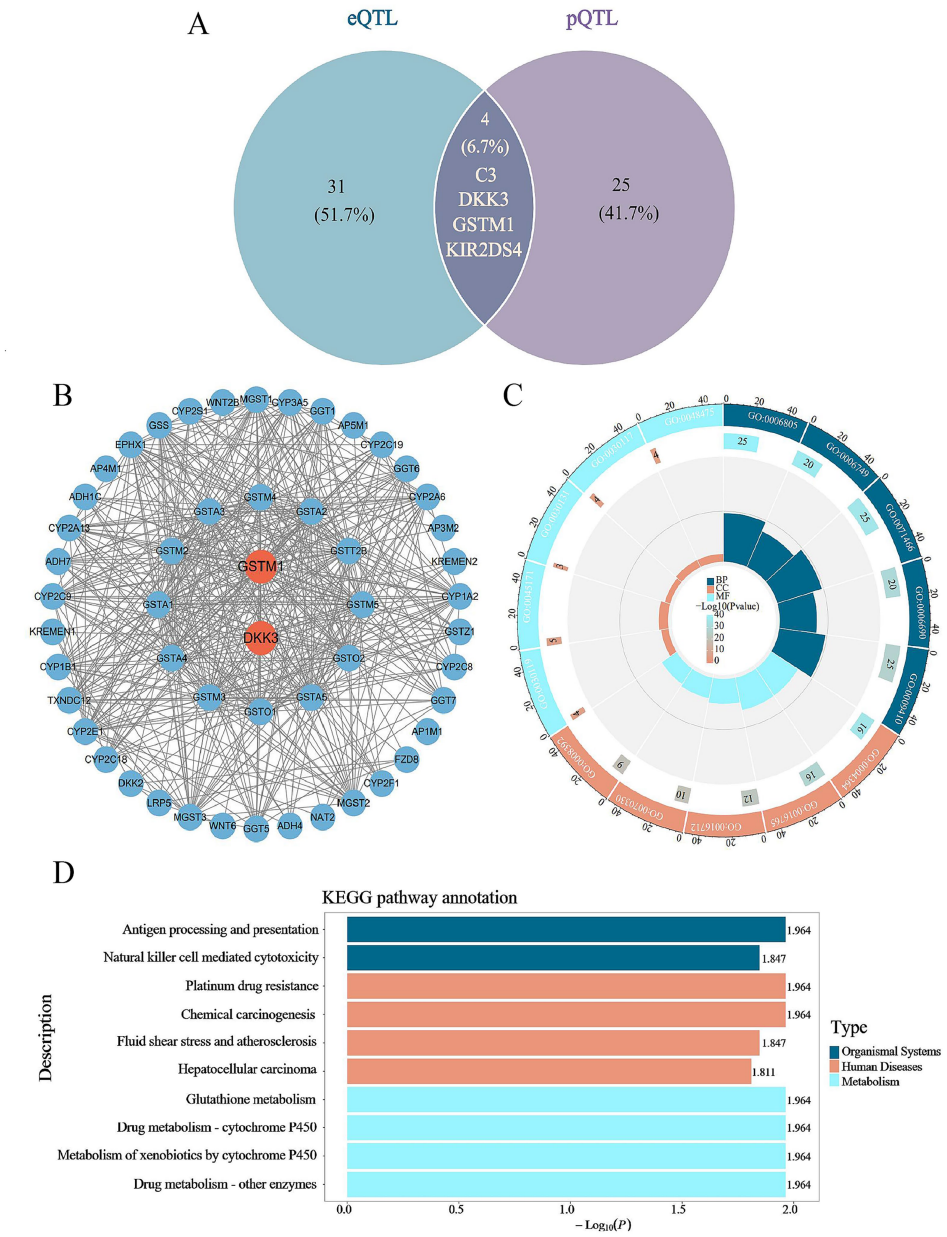
Multi-omics analysis of druggable genes and gene enrichment analysis. **(A)** This figure presents an integrative analysis of druggable genes associated with cataract using multi-omics data. Candidate genes were identified based on eQTL and pQTL datasets, followed by MR analysis to assess causal associations. The Venn diagram illustrates the number of significant genes identified in each dataset and the overlap of candidate genes across different omics layers; **(B)** PPI network of druggable genes. Orange nodes represent druggable genes; blue nodes represent interacting proteins; **(C)** GO enrichment results, including biological processes (BP), molecular functions (MF), and cellular components (CC); **(D)** KEGG pathway enrichment results based on the top 10 pathways ranked by adjusted *p*-value.

To comprehensively understand the associations between druggable gene regulation and cataract across different levels, we integrated overlapping genes identified in both eQTL and pQTL analyses and found that only four genes were strongly associated with cataract (Figure 3A; Supplementary Table S7). Among the four eQTL-identified genes, two were protective: GSTM1 (OR = 0.958, 95% CI: 0.944–0.973, *p* < 0.001) and KIR2DS4 (OR = 0.967, 95% CI: 0.954–0.981, *p* < 0.05), while the remaining two—DKK3 (OR = 1.063, 95% CI: 1.042–1.085, *p* < 0.001) and C3 (OR = 1.339, 95% CI: 1.200–1.495, *p* < 0.001)—were identified as risk genes.

Among the four genes identified in the pQTL analysis, three proteins were found to be protective factors: GSTM1 (OR = 0.957, 95% CI: 0.943–0.972, *p* < 0.001), KIR2DS4 (OR = 0.958, 95% CI: 0.942–0.974, *p* < 0.001), and C3 (OR = 0.667, 95% CI: 0.580–0.766, *p* < 0.001). DKK3 protein was identified as a risk factor (OR = 1.089, 95% CI: 1.049–1.131, *p* < 0.05).

To ensure consistency in the causal direction across multi-omics analyses, we excluded C3 due to its discordant effects, and ultimately prioritized three genes with converging multi-omics evidence: DKK3, GSTM1, and KIR2DS4.

### 3.4 Phenome-wide association analysis

There was no significant association between GSTM1 and any other gene-level traits (*p* > 5 × 10^−8^), implying that the potential adverse effects of drugs targeting this gene and the probability of horizontal pleiotropy are likely to be minor, which further bolsters the reliability of our findings. For DKK3, the PheWAS analysis revealed no significant associations with other gene-level traits, apart from those related to DKK3 protein expression (Table 2; Supplementary Figure S4), which visualizes the distribution of PheWAS associations across binary and continuous phenotypes. In addition, no associated phenotypes were identified for the KIR2DS4 gene.

**Table 2:** Traits significantly associated with the DKK3 Gene identified by PheWAS analysis.

Phenotype	Collapsing model	*p* value	No. samples	Effect size
DKK3 protein	UR	1.09724E-09	47,120	−1.60
DKK3 protein	URmtr	1.70181E-09	47,120	−1.65
DKK3 protein	flexdmg	1.97708E-14	47,120	−1.30
DKK3 protein	flexnonsynmtr	2.77301E-08	47,120	−0.64
DKK3 protein	ptv	1.09981E-13	47,120	−1.71
DKK3 protein	ptv5pcnt	1.09981E-13	47,120	−1.71
DKK3 protein	ptvraredmg	1.97708E-14	47,120	−1.30

### 3.5 Construction of the PPI network

We uploaded the three drug target genes into the STRING database to construct a PPI network. Among them, no associated proteins were identified for the druggable gene KIR2DS4. As shown in Figure 3B, the resulting interaction pathway consists of 52 protein nodes and 496 edges, with an average node degree of 19.077. The results indicate that GSTM1 and DKK3 occupy central positions in the interaction network, engaging in extensive interactions with other proteins. Notably, GSTM1 exhibited the highest interaction degree, suggesting that it may serve as a key regulatory factor within the differentially expressed gene network associated with cataract.

### 3.6 Enrichment analysis

We performed enrichment analysis on 52 genes, including two druggable genes and their top interacting proteins. Overall, 137 GO terms were enriched, distributed as 81 in biological processes (BP), 50 in molecular functions (MF), and 6 in cellular components (CC). The BP were mainly associated with xenobiotic metabolic process (GO:0006805), glutathione metabolic process (GO:0006749), and cellular response to external stimulus (GO:0071466). In terms of CC, significant enrichment was observed in AP-type membrane coat adaptor complex (GO:0030119), intercellular bridge (GO:0045171), and clathrin adaptor complex (GO:0030131). The key MF included glutathione transferase activity (GO:0004364) and oxidoreductase activity (Figure 3C).

A KEGG pathway enrichment analysis was conducted on the previously mentioned genes to investigate potential therapeutic pathways related to cataract drugs. The enrichment of 11 KEGG signaling pathways was determined by adjusted *p*-values, and the top 10 pathways were shown in a bar chart. As depicted in Figure 3D, these genes are largely associated with multiple signaling pathways, including the processing and presentation of antigens, cytotoxicity mediated by NK cells, and pathways involved in drug metabolism.

### 3.7 GeneMANIA analysis of druggable genes

GeneMANIA analysis was performed on the three druggable genes. As shown in the resulting network (Supplementary Figure S5), a total of 20 related genes were identified. The related biological roles involved the metabolic process of glutathione derivatives, binding of oligopeptides, and transferase activity that transfers alkyl or aryl groups (excluding methyl groups), peptide binding, modified amino acid metabolic process, sulfur compound biosynthetic process, and antigen processing and presentation of endogenous peptide antigen.

### 3.8 Predicting candidate drugs and performing molecular docking

**Figure 4:**
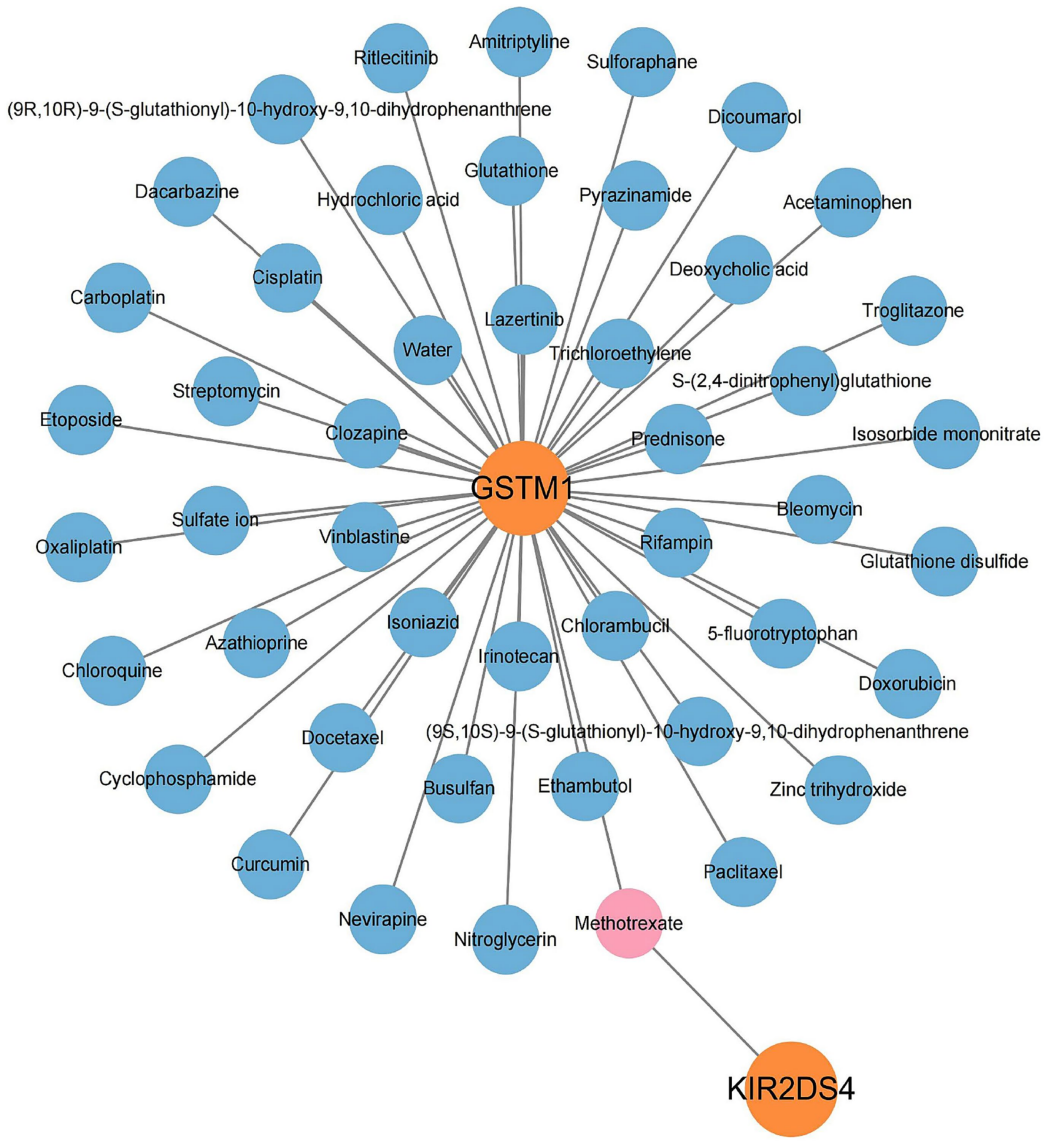
Drug prediction for candidate druggable genes. A total of 48 drugs were predicted based on GeneCards database queries for *GSTM1* and *KIR2DS4*; no candidate drugs were identified for *DKK3*.

The GeneCards database was utilized to forecast possible therapeutic agents aimed at the candidate genes. No potential drugs were identified for DKK3. In contrast, GSTM1 was predicted to interact with 48 drugs, and KIR2DS4 with one drug. In total, 48 candidate drugs were identified for the two druggable genes (Figure 4).

**Figure 5:**
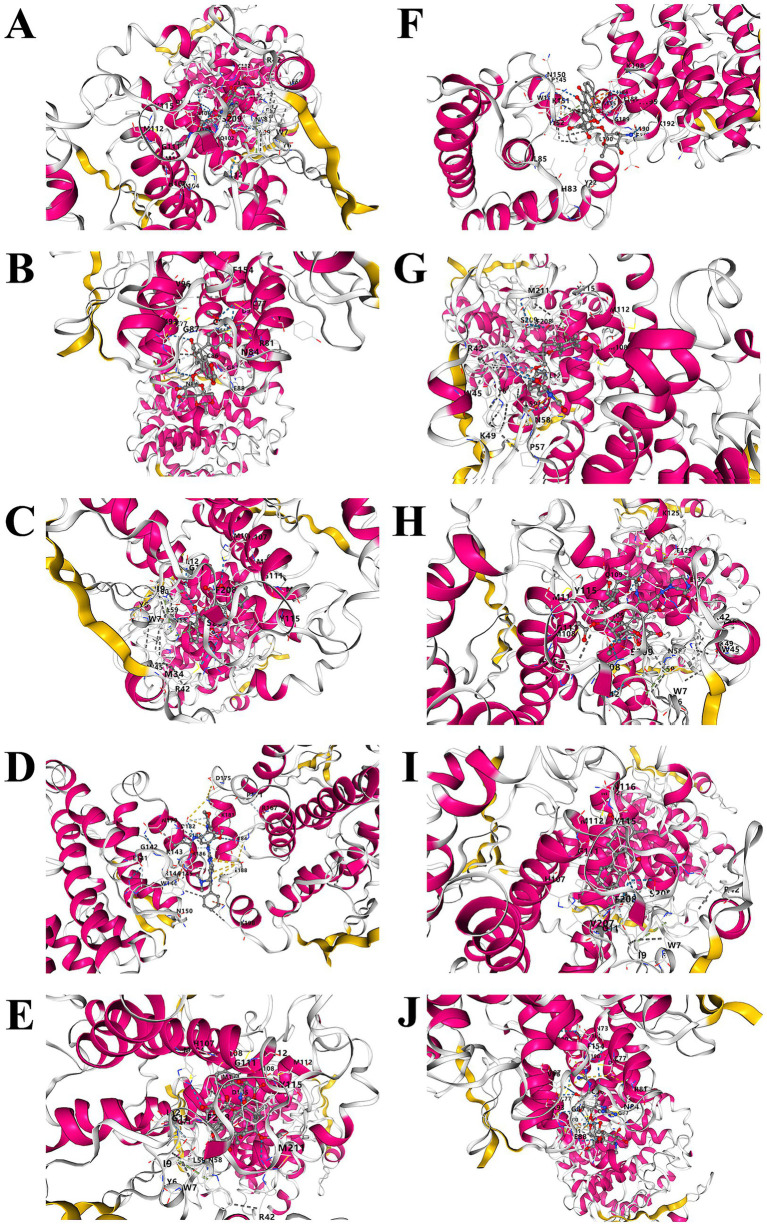
Molecular docking interactions between GSTM1 and the top 10 candidate drugs ranked by binding energy. Docking results are shown for: **(A)** Irinotecan; **(B)** Etoposide; **(C)** Dicoumarol; **(D)** Lazertinib; **(E)** Paclitaxel; **(F)** Doxorubicin; **(G)** Troglitazone; **(H)** Rifampin, **(I)** Deoxycholic acid; **(J)** Streptomycin; compounds are ordered from lowest to highest binding energy, indicating increasing docking energy and decreasing binding stability.

In this study, 48 candidate drugs were selected for further analysis. Among them, four lacked three-dimensional structural information, and five exhibited ligand transfer errors (including Methotrexate predicted to target KIR2DS4), rendering them unsuitable for docking. The remaining drugs were successfully visualized (Table 3; Supplementary Table S8). Using AutoDock, we analyzed the binding sites and interactions between the GSTM1 protein and 38 candidate compounds. The results indicated that GSTM1 could form stable interactions with 30 drugs, including Curcumin, Amitriptyline, Glutathione disulfide (GSSG), and Chloroquine, with representative docking conformations shown in Supplementary Figures S6 and S7. Binding energies were calculated for each protein–ligand interaction. As shown in Figure 5, the top ten compounds with the lowest binding energies were identified, with the strongest interaction observed between GSTM1 and Irinotecan (−10.4 kcal/mol), indicating a stable binding affinity.

**Table 3:** Identification of druggable genes and actionable compounds.

Druggable gene	Molecule type	Compounds	Action type	Clinical development activities	Druggable gene molecular function
GSTM1	Protein	Curcumin	Inhibitor	Approved, Investigational; Tyrosinase inhibitor;	Conjugation of reduced glutathione to a wide number of exogenous and endogenous hydrophobic electrophiles. Involved in the formation of glutathione conjugates of both PGA2 and PGJ. Participates in the formation of novel hepoxilin regioisomers.
		Amitriptyline	Inhibitor	Approved; Serotonin /norepinephrine receptor/5-HT4/5-HT2 inhibitor, Small Molecule, Antidepressive Agents, Tricyclic, 5-HT and noradrenalin re-uptake inhibitor; also TrkA/B agonist;	
		Glutathione disulfide	Activator	Approved, Experimental, Investigational;	
		Chloroquine	Inhibitor	Approved, Investigational, Vet_approved;	
DKK3	Protein	**–**	**–**	**–**	Antagonizes canonical Wnt signaling by inhibiting LRP5/6 interaction with Wnt and by forming a ternary complex with the transmembrane protein KREMEN that promotes internalization of LRP5/6. DKKs play an important role in vertebrate development, where they locally inhibit Wnt regulated processes such as antero-posterior axial patterning, limb development, somitogenesis and eye formation. In the adult, Dkks are implicated in bone formation and bone disease, cancer and Alzheimer disease.
KIR2DS4	Protein	**–**	**–**	**–**	Receptor on NK cells for HLA-C alleles. Does not inhibit the activity of NK cells.

PGA2, prostaglandin A2; PGJ2, prostaglandin J2; NK, natural killer cells.

Preclinical or clinical development activity of the three candidate druggable genes for cataract was further evaluated (Table 3). It was found that Curcumin, Amitriptyline, GSSG, and Chloroquine, which are associated with the GSTM1 gene, have been assessed in clinical trials for other diseases, though not yet for cataract. These drugs demonstrate potential therapeutic value through modulation of GSTM1 expression or the oxidative stress pathway. However, their specific mechanisms and target selectivity require further elucidation.

### 3.9 Effects of oxidative stress on the proliferation, apoptosis, and hub gene expression in lens epithelial cells

**Figure 6:**
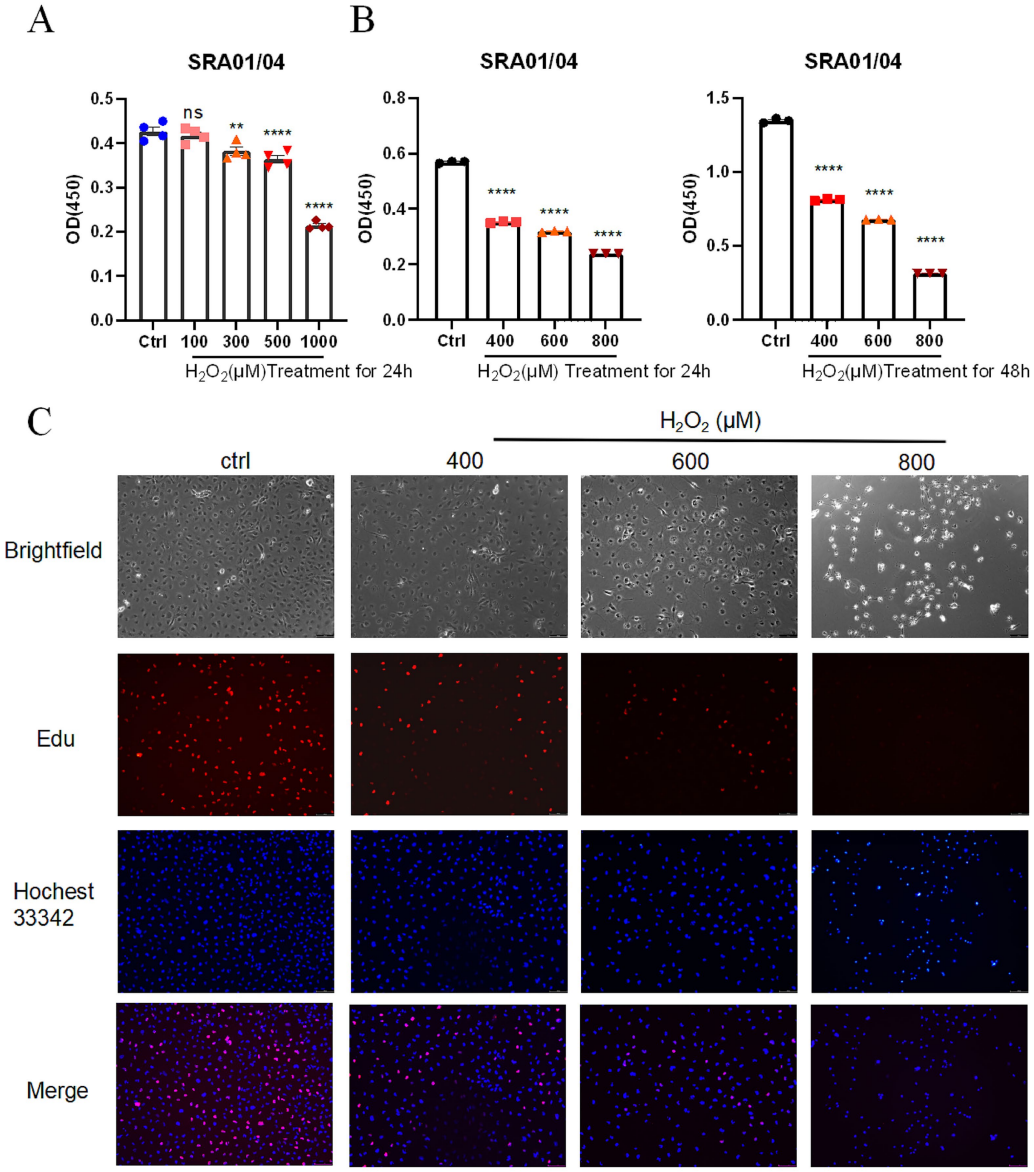
Effect of hydrogen peroxide on the proliferation of SRA01/04 corneal epithelial cells as assessed by CCK8 and EdU assays. **(A)** CCK8 assay showing the cell viability after 24 h of treatment with 100, 300, 500, and 1,000 μM H_2_O_2_. **(B)** CCK8 assay showing the cell viability after 24 and 48 h of treatment with 400, 600, and 800 μM H_2_O_2_. **(C)** EdU assay results showing the proportion of EdU-positive cells in response to 24 h H_2_O_2_ treatment. *p* < 0.01, ^***^p < 0.001, ^****^p < 0.0001.

**Figure 7:**
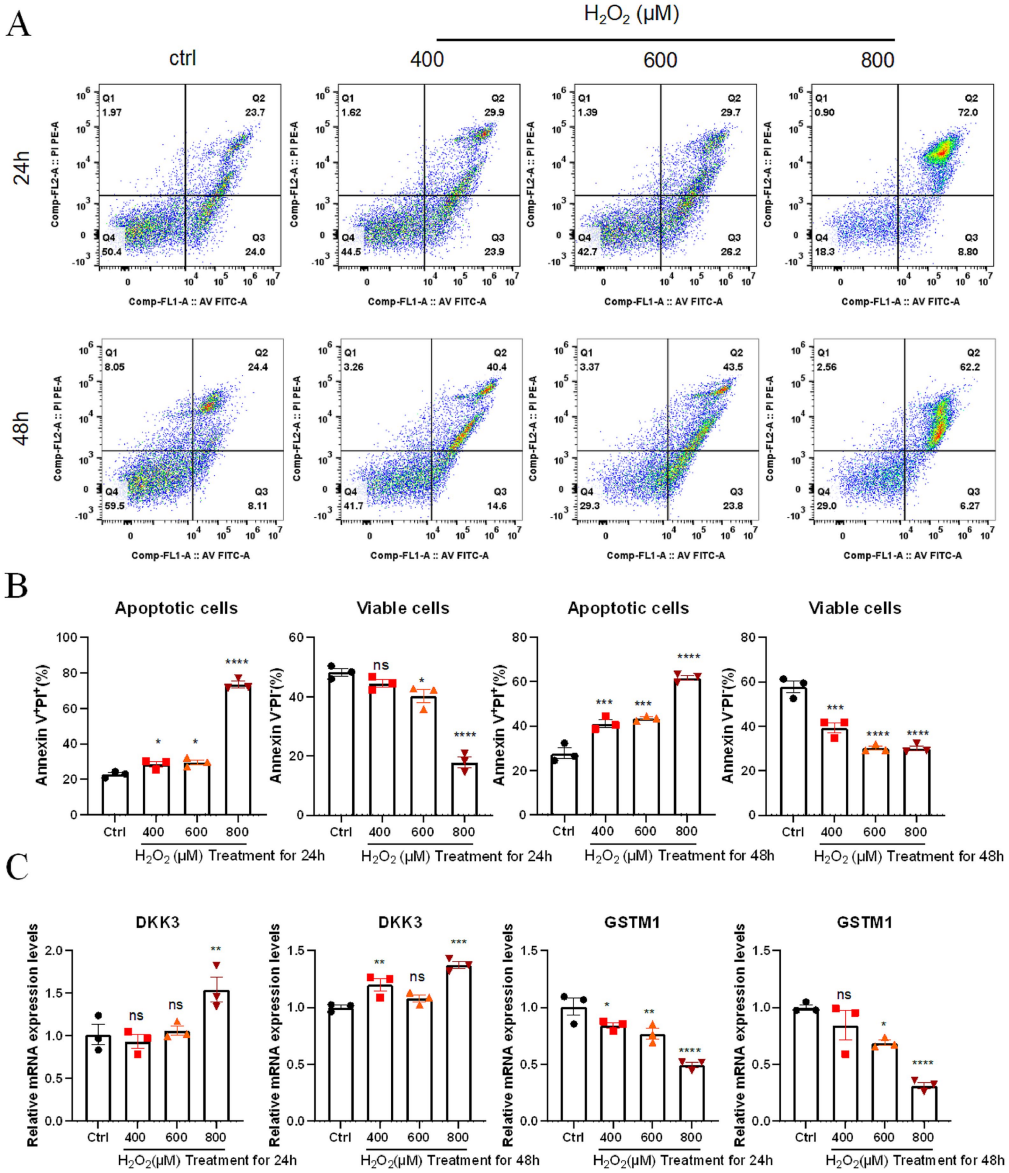
Effect of hydrogen peroxide on apoptosis and the expression of key genes in SRA01/04 lens epithelial cells. **(A)** Flow cytometry scatter plot showing the apoptotic populations after 24 and 48 h of H_2_O_2_ treatment. **(B)** Statistical analysis showing the dose-dependent increase in apoptosis, with Annexin V^+^/PI^+^ cells representing apoptotic cells and Annexin V^−^/PI^−^ cells representing live cells. **(C)** qPCR analysis of *DKK3* and *GSTM1* mRNA expression in SRA01/04 lens epithelial cells after 24 and 48 h of H_2_O_2_ treatment. ^*^p < 0.05, ^**^p < 0.01, ^***^p < 0.001, ^****^p < 0.0001.

After treatment of SRA01/04 lens epithelial cells with 100–1,000 μM H_2_O_2_ for 24 h, CCK8 assay results showed that cell viability significantly decreased at 300 μM (*p* < 0.01) and was further reduced at 500 and 1,000 μM (*p* < 0.0001), demonstrating a clear concentration-dependent trend (Figure 6A). When the concentrations were set at 400, 600, and 800 μM for 24 h and 48 h, cell viability consistently decreased in a dose-dependent manner under both conditions (*p* < 0.0001) (Figure 6B). EdU assay results indicated that the proportion of EdU-positive cells declined with increasing H_2_O_2_ concentrations (Figure 6C), suggesting that DNA synthesis activity and proliferation were markedly inhibited. This trend was consistent with the CCK8 results. In addition, flow cytometry analysis revealed a dose-dependent increase in apoptosis following H_2_O_2_ exposure, with the proportion of Annexin V^+^/PI^+^ cells reaching the highest level at 800 μM (*p* < 0.0001), and this effect was more pronounced in the 48 h group (Figures 7A,B). Collectively, these results demonstrate that H_2_O_2_ stimulation suppresses the proliferation of SRA01/04 lens epithelial cells and significantly promotes apoptosis in a dose-dependent manner.

From the previous findings, DKK3 and GSTM1 were identified as key genes associated with cataracts. Therefore, we further investigated the effects of H_2_O_2_ on the expression of these cataract-related genes in SRA01/04 lens epithelial cells. The results showed that 800 μM H_2_O_2_ significantly upregulated DKK3 expression, reaching statistical significance at both 24 h and 48 h (*p* < 0.01, *p* < 0.001) (Figure 7C). In contrast, GSTM1 expression declined with increasing H_2_O_2_ concentrations under both time conditions, with significant suppression observed at 800 μM (*p* < 0.0001) (Figure 7C). These findings indicate that H_2_O_2_ treatment promotes the expression of DKK3 while inhibiting the expression of GSTM1 in SRA01/04 lens epithelial cells. These findings are consistent with the direction of the causal effects inferred from the MR analysis, which suggested that increased DKK3 expression is associated with higher cataract risk, whereas higher GSTM1 expression is protective. Under oxidative stress conditions, the observed upregulation of DKK3 and downregulation of GSTM1 were accompanied by reduced cell proliferation and increased apoptosis, providing biological consistency with the genetic evidence obtained from MR analysis.

## 4 Discussion

This study, based on multi-omics MR analysis, integrated druggable gene targets with cataract GWAS data and identified three druggable genes significantly associated with cataracts (DKK3, GSTM1, KIR2DS4). Further systematic evaluation of their potential functions was conducted through Phe-WAS, enrichment analysis, GeneMANIA prediction, and PPI network construction, while drug prediction and molecular docking analyses were employed to explore their clinical development value. The results showed that only GSTM1 yielded effective molecular docking outcomes, suggesting it may represent the most promising therapeutic target for cataract intervention. Finally, *in vitro* experiments examined the effects of oxidative stress on the proliferation, apoptosis, and expression of key genes in SRA01/04 lens epithelial cells. Under oxidative stress conditions, GSTM1 expression decreased while DKK3 expression increased, which was consistent with the MR results indicating GSTM1 as a protective factor and DKK3 as a risk-associated gene for cataract. Oxidative stress was selected as the experimental model because it is widely recognized as a central mechanism in cataract development and can directly disrupt lens epithelial cell homeostasis.

As an important component of reactive oxygen species, H_2_O_2_, at low levels, can maintain cellular homeostasis by regulating redox signaling (26). However, excessive accumulation of H_2_O_2_ leads to oxidative damage to proteins, lipids, and DNA, which in turn activates inflammatory responses and results in cellular dysfunction (27, 28). In terms of cell fate regulation, H_2_O_2_ directly impacts the balance between cell proliferation and apoptosis. High concentrations of H_2_O_2_ can promote caspase family activation through mitochondrial pathways or p53-dependent mechanisms, inducing apoptosis or even necrosis (29). Previous studies have reported that excessive H_2_O_2_ induces apoptosis in lens epithelial cells and promotes protein cross-linking and aggregation, accelerating cataract formation (30). In this study, we similarly observed that H_2_O_2_ treatment led to a decrease in SRA01/04 lens epithelial cell viability, inhibited proliferation, and significantly increased apoptosis levels. This is consistent with clinical findings showing a marked increase in H_2_O_2_ levels in cataractous lens tissues, further supporting the central role of oxidative stress in cataract pathogenesis. Although antioxidants have demonstrated protective effects in animal and cell models, their clinical efficacy remains unstable (31), suggesting the need to explore new molecular targets to improve prevention and treatment strategies.

Among the candidate target genes, GSTM1, a member of the glutathione S-transferase (GST) family, is located on human chromosome 1 and is an essential component of cellular antioxidant defense. Its primary function is to catalyze the conjugation of glutathione with electrophilic substances, facilitating the removal of harmful metabolic products, thereby alleviating oxidative stress damage (32–34). Several studies have shown a close association between GSTM1 deletion and an increased risk of cataracts, with the underlying mechanism being linked to a decline in lens antioxidant capacity (35). For instance, Gericke et al. found that GSTM1 deletion significantly reduced the ability of cells to respond to oxidative damage, accelerating lens cell degeneration (36). Ellwanger et al. further pointed out that individuals with GSTM1 deletion are more prone to lens structural abnormalities under oxidative stress conditions (32). The results of this study are consistent with these findings, as GSTM1 expression was significantly suppressed in the H_2_O_2_-induced oxidative stress model, while the expression of another candidate gene, DKK3, was upregulated. This phenomenon suggests that GSTM1 may exert a protective effect by maintaining the redox homeostasis of the lens. Its deletion or insufficient expression may lead to an imbalance in antioxidant defense, thereby increasing cataract susceptibility.

Recent multi-omics studies have emphasized the role of transcriptional regulatory networks and mitochondrial stress responses in oxidative stress–related diseases (37, 38). For example, Li et al. showed that integrated multi-omics analyses can identify transcription factor–mediated regulatory networks linking oxidative stress to inflammatory and metabolic processes. In addition, She et al. reported that mitochondrial dysfunction and stress-responsive signaling pathways may act as central regulators connecting redox imbalance with cellular injury. These findings provide additional context for the results of the present study. GSTM1 is a key enzyme in glutathione-mediated detoxification and plays an important role in maintaining intracellular redox homeostasis. Reduced GSTM1 expression may impair antioxidant defense and increase oxidative stress in lens epithelial cells. In contrast, stress-induced upregulation of DKK3 may influence signaling pathways related to cellular stress responses and apoptosis. Together, these observations suggest that GSTM1 and DKK3 may contribute to cataract development through oxidative stress–related regulatory networks involving transcriptional regulation and mitochondrial stress signaling, while KIR2DS4 may represent an additional immune-related factor that warrants further investigation.

Among the drugs predicted to target GSTM1, curcumin appears to be the most promising. Existing studies have shown that curcumin can effectively delay cataract formation by scavenging free radicals and inhibiting oxidative stress, demonstrating potential as an anti-cataract agent (39). In the lens antioxidant system, reduced glutathione (GSH) exerts a protective effect by directly scavenging free radicals, while the accumulation of GSSG reflects an increased oxidative burden (40). GSTM1 regulates glutathione metabolism, further affecting the lens’s resistance to oxidative damage, and is closely associated with cataract development (40, 41). Additionally, chloroquine, a traditional antimalarial drug, is used in the treatment of immune-related diseases due to its anti-inflammatory effects (42), and may play a role in modulating the ocular microenvironment and delaying cataract progression. In contrast, the use of antidepressants such as amitriptyline has been associated with an increased risk of cortical cataracts (43). Although research on these drugs is still in its early stages with insufficient clinical evidence, the polymorphic characteristics of the GSTM1 gene may provide a foundation for developing personalized drug intervention strategies, potentially offering new directions for cataract prevention and treatment in the future.

In addition to GSTM1, this study also identified DKK3 as an important candidate gene associated with cataracts. DKK3, a member of the Dickkopf family, plays a crucial role in regulating the differentiation and apoptosis of lens epithelial cells. By inhibiting the Wnt signaling pathway, it may influence the maintenance of lens transparency (44, 45). Previous studies have found that abnormal expression of DKK3 is closely associated with lens dysfunction, suggesting its potential role in cataract development (46, 47). In this study, H_2_O_2_ stimulation led to a significant upregulation of DKK3 expression in SRA01/04 cells, exhibiting a time-dependent trend. This result is consistent with our previous MR analysis, suggesting that DKK3 may accelerate cataract development under oxidative stress by promoting apoptosis or regulating signal transduction. Although preliminary evidence has revealed an association between DKK3 and cataracts, its specific molecular mechanisms remain unclear. Further systematic exploration, integrating genetic and *in vitro* experimental evidence, is required to better define its value as a cataract risk gene and a potential therapeutic target.

As a member of the killer immunoglobulin-like receptor family, KIR2DS4 is an activating receptor on NK cells that modulates their cytotoxic response (48–50). Although KIR2DS4 plays an important role in immune regulation, no studies have directly revealed its association with cataracts. Because KIR2DS4 is primarily expressed in immune cells such as natural killer cells, whereas the SRA01/04 cell line used in this study represents lens epithelial cells, it was not included in the in vitro validation experiments. Therefore, whether KIR2DS4 can be considered a druggable gene for cataracts requires further investigation in more appropriate experimental models. From a translational perspective, GSTM1 may represent a more feasible target for early therapeutic exploration, particularly through drug repurposing strategies aimed at modulating oxidative stress pathways. However, further validation in animal models and carefully designed clinical studies will be required to evaluate the safety and efficacy of targeting these genes in cataract treatment.

## 5 Strengths and limitations

This study has several advantages in the identification and validation of druggable genes for cataracts. First, we focused on candidate genes supported by existing functional or disease association evidence and enhanced the robustness and clinical relevance of the results by integrating transcriptomic, proteomic, and cataract GWAS data. Second, the use of cis-acting variants as instrumental variables provided a clearer insight into the potential causal relationship between gene expression and disease risk. Additionally, multiple statistical corrections reduced the risk of false positives. PheWAS further assessed the systemic safety of potential targets, offering valuable references for drug development. Furthermore, this study goes beyond computational analysis by integrating in vitro experiments (CCK8, EdU, flow cytometry, and qPCR), systematically validating the effects of oxidative stress on the expression of key cataract genes (DKK3 and GSTM1) and the biological behavior of lens epithelial cells, thereby enhancing the biological credibility and translational significance of the findings.

Despite these findings, this study has several limitations. First, the GWAS data were derived mainly from European populations, which may limit the generalizability of the results to other ethnic groups. This restriction largely reflects the current availability of large-scale cataract GWAS datasets rather than a specific design choice of the present study; therefore, validation in more diverse populations is needed. Second, the eQTL and pQTL datasets used in this study were derived from peripheral blood and may not fully reflect tissue-specific regulatory mechanisms in lens and ocular tissues. However, previous studies have shown that many cis-eQTL signals are shared across multiple tissues, particularly for genes involved in systemic biological processes such as oxidative stress, immune regulation, and metabolism. Third, molecular docking was used as a computational screening approach to explore potential drug–target interactions. However, docking scores alone cannot directly reflect biological activity, and the results may be influenced by the accuracy of protein structures and ligand conformations. Therefore, these predicted interactions should be interpreted cautiously and require further validation through biochemical or cell-based experiments. In addition, advanced analytical methods, such as electrochemical sensing technologies, may help experimentally validate predicted molecular interactions in future studies (51).

## 6 Conclusion

This study identified three candidate genes (DKK3, GSTM1, and KIR2DS4) associated with cataract by integrating eQTL and pQTL datasets with Mendelian randomization analysis. Among them, GSTM1 showed stable interactions with multiple compounds in molecular docking analyses, suggesting potential druggability. *In vitro* experiments further demonstrated that H_2_O_2_-induced oxidative stress inhibited the proliferation of lens epithelial cells and promoted apoptosis, accompanied by increased DKK3 expression and decreased GSTM1 expression. These findings provide genetic evidence supporting the potential involvement of these genes in cataract development, while the in vitro results offer additional biological support under oxidative stress conditions. However, these results should not be interpreted as direct evidence of therapeutic efficacy. Further mechanistic studies, animal experiments, and clinical investigations are required to determine whether targeting these genes could have potential clinical applications in cataract prevention or treatment.

## Data Availability

The original contributions presented in the study are included in the article/Supplementary material, further inquiries can be directed to the corresponding author.

## Footnotes

1
https://www.dgidb.org/downloads

2
https://www.eqtlgen.org/phase1.html

3
https://registry.opendata.aws/ukbppp/

4
https://azphewas.com

5
https://string-db.org/

6
https://www.genecards.org

7
https://cadd.labshare.cn/cb-dock2/php/index.php

## Glossary

MR: Mendelian randomization
eQTLs: expression quantitative trait loci
pQTLs: protein quantitative trait loci
GWAS: genome-wide association studies
DGIdb: Drug–Gene Interaction Database
UKB-PPP: UK Biobank Pharma Proteomics Project
IVs: Eligible instrumental variables
IVW: inverse-variance weighted
WME: weighted median estimator
PheWAS: phenome-wide association studies
PPI: protein–protein interaction
GO: Gene Ontology
KEGG: Kyoto Encyclopedia of Genes and Genomes
HGNC: HUGO Gene Nomenclature Committee
BP: biological processes
MF: molecular functions
CC: cellular components
GST: glutathione S-transferase
GSH: Glutathione
GSSG: glutathione disulfide
NK: natural killer
KIR: killer cell immunoglobulin-like receptor.
