# Supplementary material for: Identification and validation of druggable targets for cataract using mendelian randomization: functional insights from multi-omics and an oxidative stress model
Source: Front Med (Lausanne). 2026 Apr 16;13:1741371. doi: 10.3389/fmed.2026.1741371 (PMC13128393; doi:10.3389/fmed.2026.1741371)
Supplement: Supplementary file 1 [file Data_sheet_1.pdf]

Identification and Validation of Druggable Targets for Cataract Using Mendelian Randomization: Functional Insights from Multi-Omics and an Oxidative Stress Model

Min Lin[1,*] Jie Zeng[2]

[1]Department of Ophthalmology, Fujian Provincial Geriatric Hospital, Fujian Provincial Hospital North Branch, Fuzhou, China.

[2]Department of Ophthalmology, Fujian Medical University Affiliated Min Dong Hospital, Ningde, China.

***** Corresponding.

Postal address:Department of Ophthalmology, Fujian Provincial Geriatric Hospital,147 North Second Ring Middle Road, Gulou District, Fuzhou, 350001, Fujian, China.

Email: linmin_fjmu@163.com

Tel: (86) 13774622005

Supplementary Figure Legends

Figure S1. Flowchart of the MR Study Design.

Figure S2.Leave-one-out analysis of eQTL-based Mendelian randomization results.

Figure S3.Leave-one-out analysis of pQTL-based Mendelian randomization results

Figure S4. PheWAS analysis of DKK3 and GSTM1 with binary and continuous traits.

Figure S5. GeneMANIA network analysis of the three druggable genes.

Figure S6. Molecular docking interactions between GSTM1 and Candidate drugs ranked bybinding energy.

Figure S7. Molecular docking interactions between GSTM1 and Candidate drugs ranked by binding energy.

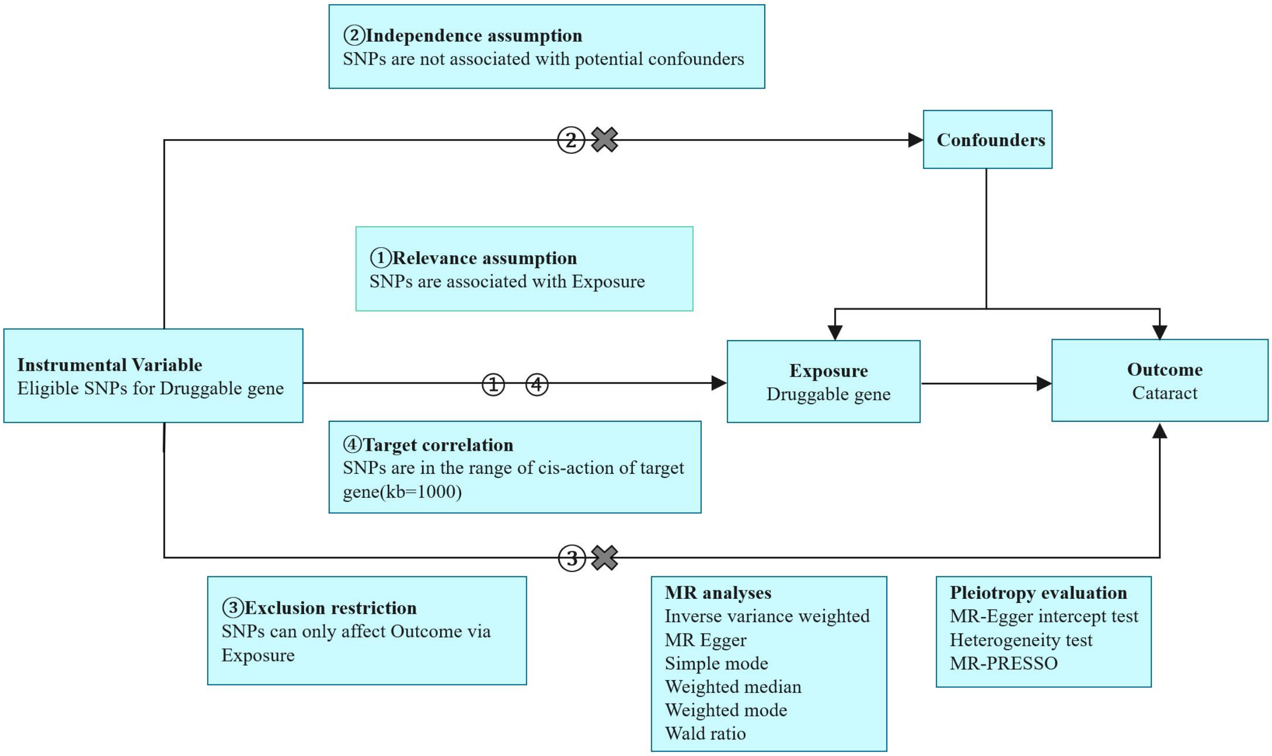

Supplementary Figure 1. Flowchart of the MR Study Design

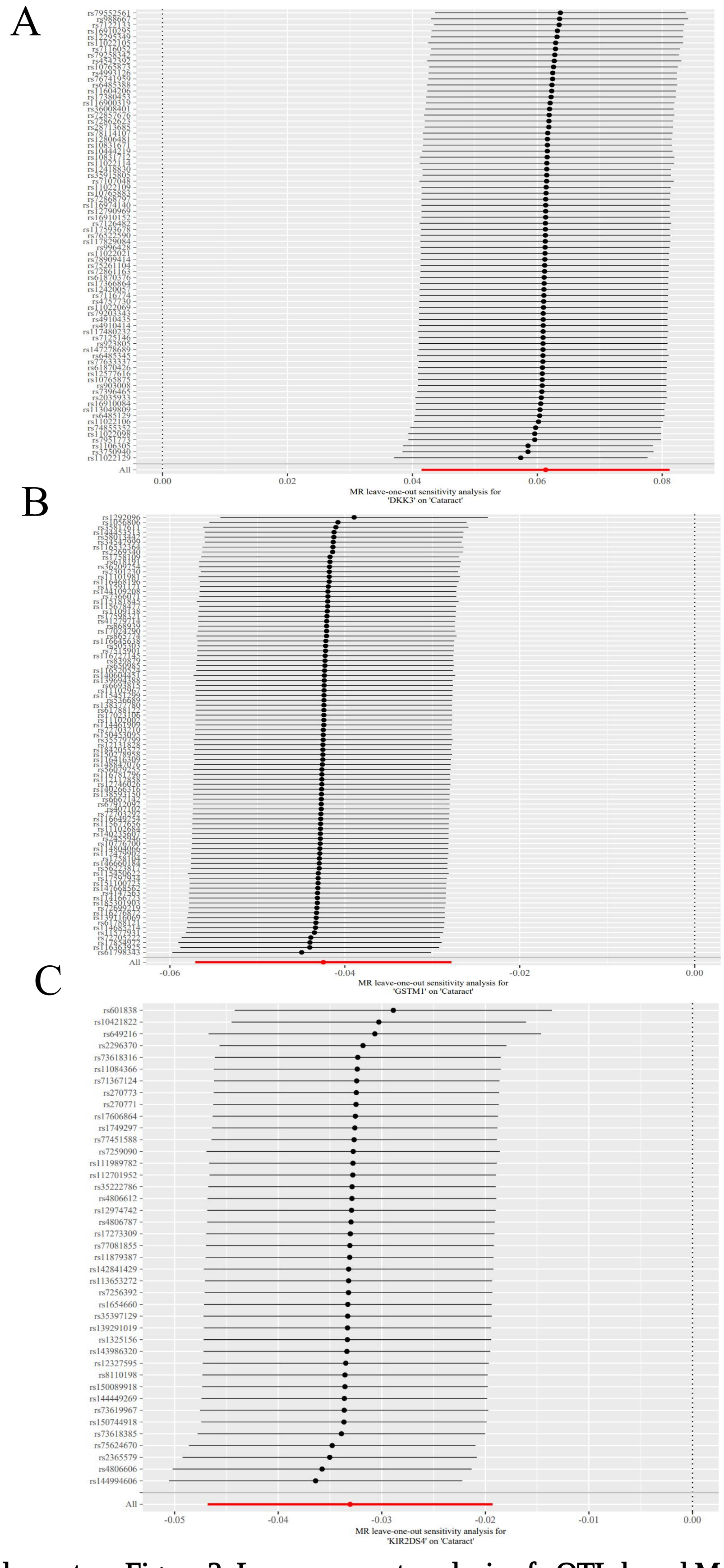

Supplementary Figure 2. Leave-one-out analysis of eQTL-based Mendelian randomization results

(A)Leave-one-out analysis of the DKK3 gene;

(B)Leave-one-out analysis of the GSTM1 gene;

(B)Leave-one-out analysis of the KIR2DS4 gene;

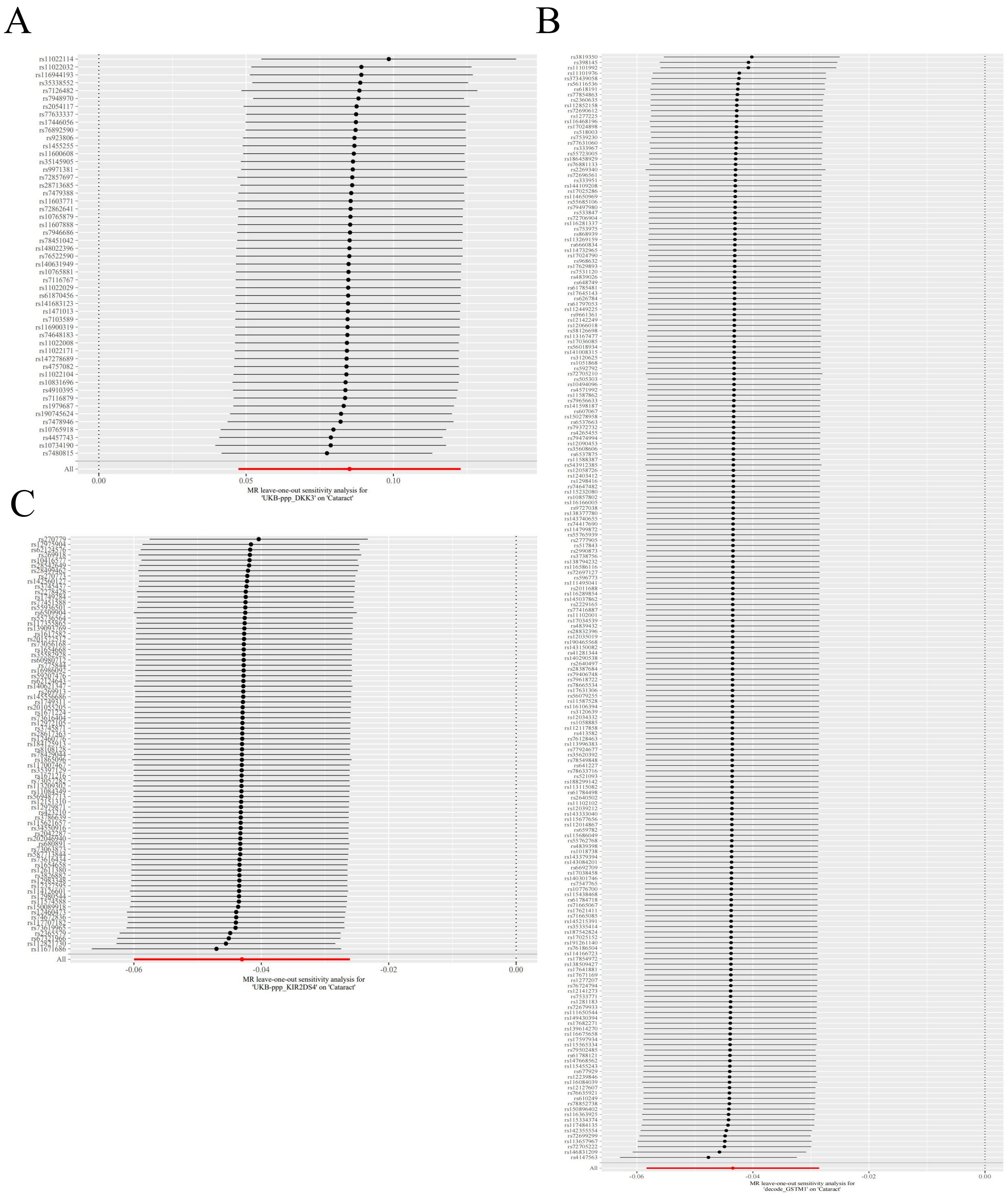

Supplementary Figure 3. Leave-one-out analysis of pQTL-based Mendelian randomization results

(A)Leave-one-out analysis of the DKK3 gene;

(B)Leave-one-out analysis of the GSTM1 gene;

(B)Leave-one-out analysis of the KIR2DS4 gene;

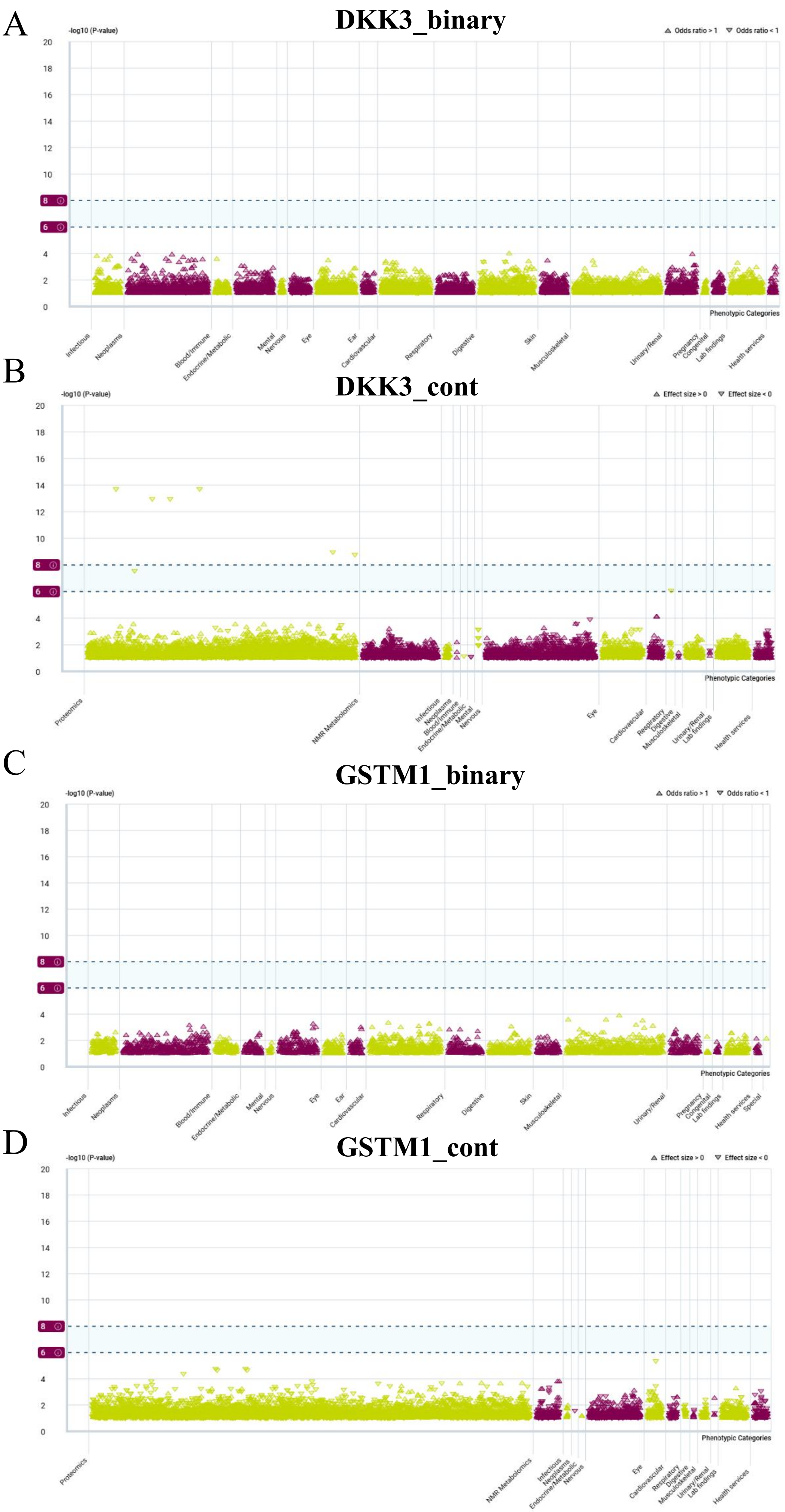
A
DKK3_binary
-log10 (P-value)
Odds ratio > 1
Odds ratio < 1
Phenotypic Categories
Infectious
Neoplasms
Blood/Immune
Endocrine/Metabolic
Mental
Nervous
Eye
Ear
Cardiovascular
Respiratory
Digestive
Skin
Musculoskeletal
Urinary/Renal
Pregnancy
Congenital
Lab findings
Health services
B
DKK3_cont
-log10 (P-value)
Effect size > 0
Effect size < 0
Phenotypic Categories
Proteomics
NMR Metabolomics
Infectious
Neoplasms
Blood/Immune
Endocrine/Metabolic
Mental
Nervous
Eye
Cardiovascular
Respiratory
Digestive
Musculoskeletal
Urinary/Renal
Lab findings
Health services
C
GSTM1_binary
-log10 (P-value)
Odds ratio > 1
Odds ratio < 1
Phenotypic Categories
Infectious
Neoplasms
Blood/Immune
Endocrine/Metabolic
Mental
Nervous
Eye
Ear
Cardiovascular
Respiratory
Digestive
Skin
Musculoskeletal
Urinary/Renal
Pregnancy
Congenital
Lab findings
Health services
Special
D
GSTM1_cont
-log10 (P-value)
Effect size > 0
Effect size < 0
Phenotypic Categories
Proteomics
NMR Metabolomics
Infectious
Neoplasms
Endocrine/Metabolic
Nervous
Eye
Cardiovascular
Respiratory
Digestive
Musculoskeletal
Urinary/Renal
Lab findings
Health services

Supplementary Figure 4. PheWAS analysis of DKK3 and GSTM1 with binary and continuous traits.(A) Associations between DKK3 and binary traits identified by PheWAS analysis; (B) Associations between DKK3 and continuous traits; (C) Associations between GSTM1 and binary traits; (D) Associations between GSTM1 and continuous traits;

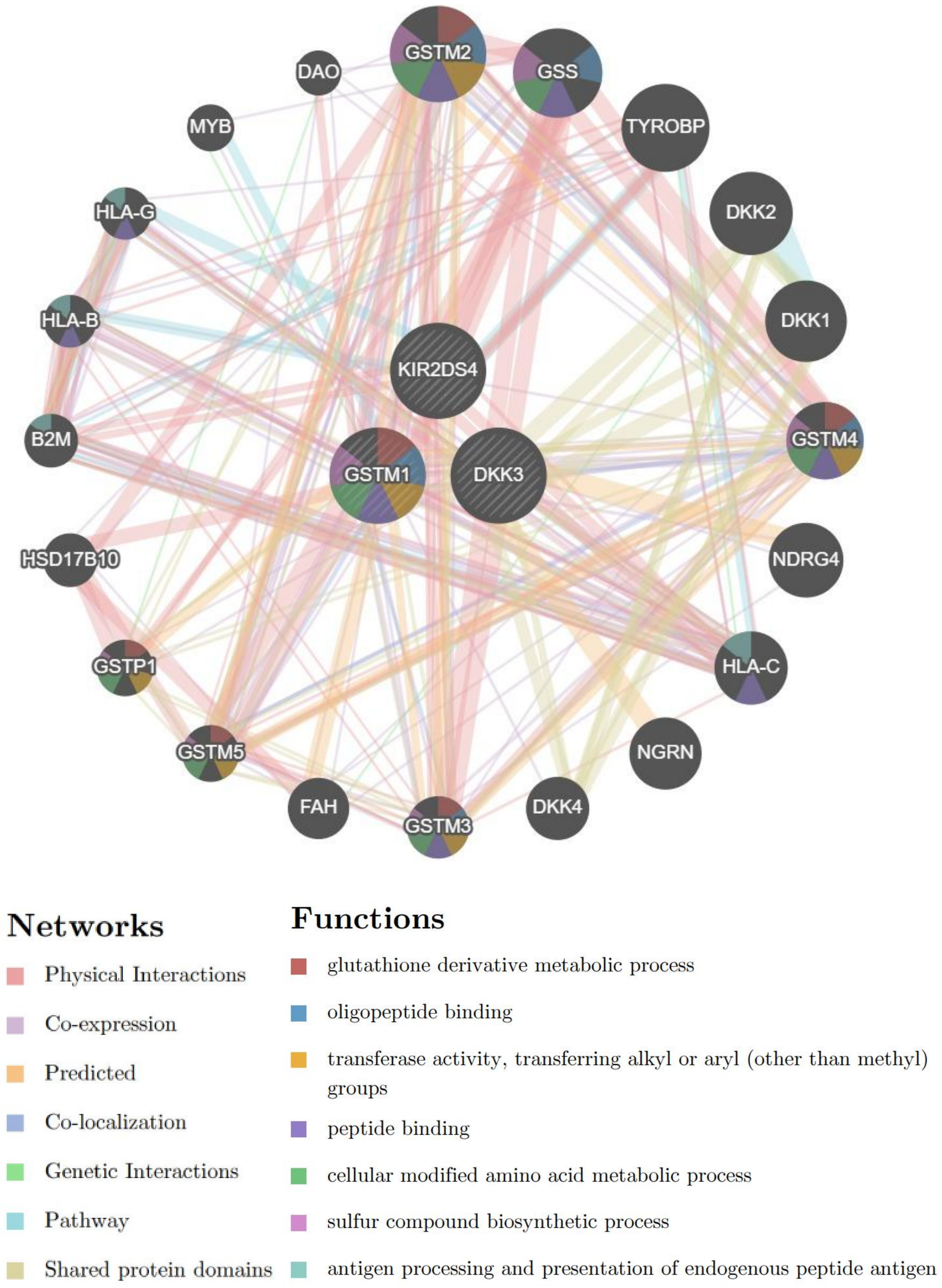

Supplementary Figure 5. GeneMANIA network analysis of the three druggable genes. This figure shows the functional interaction network of the three identified druggable genes—DKK3, GSTM1, and KIR2DS4—constructed using the GeneMANIA platform.

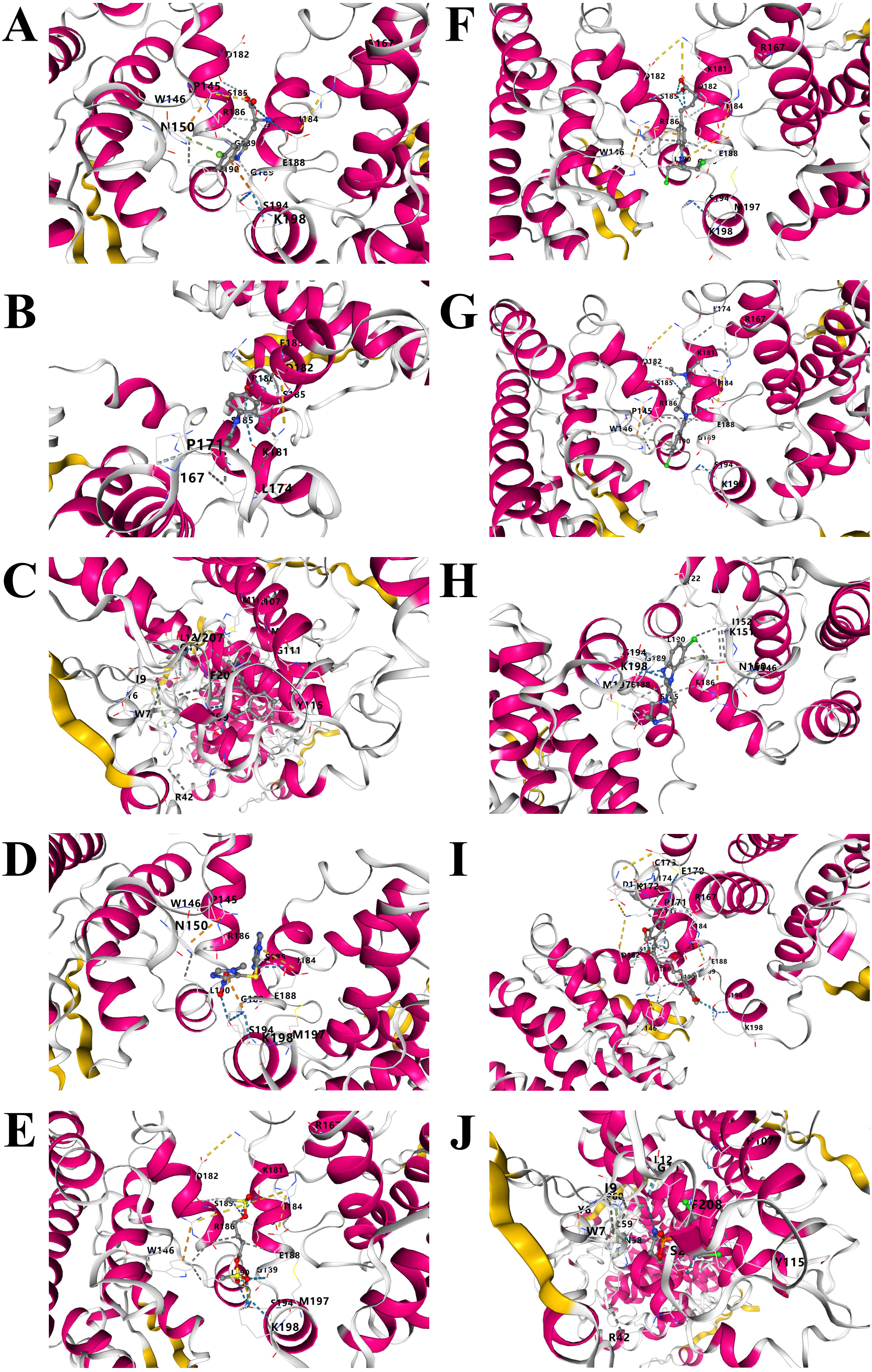

A
D182
P145
W146
S185
R186
I184
N150
E188
S194
K198
F
R167
D182
K181
S185
I184
R186
E188
W146
S194
M197
K198
B
D182
S185
P171
K181
167
L174
G
R167
K181
D182
S185
P145
R186
W146
E188
S194
C
I9
W7
F20
G111
Y115
R42
H
I152
S194
K198
R186
D
W146
P145
N150
R186
I184
E188
S194
K198
M197
I
C173
E170
R167
K198
E
R167
D182
K181
S185
R186
W146
E188
S194
M197
K198
J
L12
I9
F208
W7
Y115
R42

Supplementary Figure 6. Molecular docking interactions between GSTM1 and Candidate drugs ranked by binding energy.

Docking results are shown for:

(A) 5-fluorotryptophan;
(B) Acetaminophen;
(C) Amitriptyline;
(D) Azathioprine;
(E) Busulfan;
(F) Chlorambucil;
(G) Chloroquine;
(H) Clozapine;
(I) Curcumin;
(J) Cyclophosphamide;

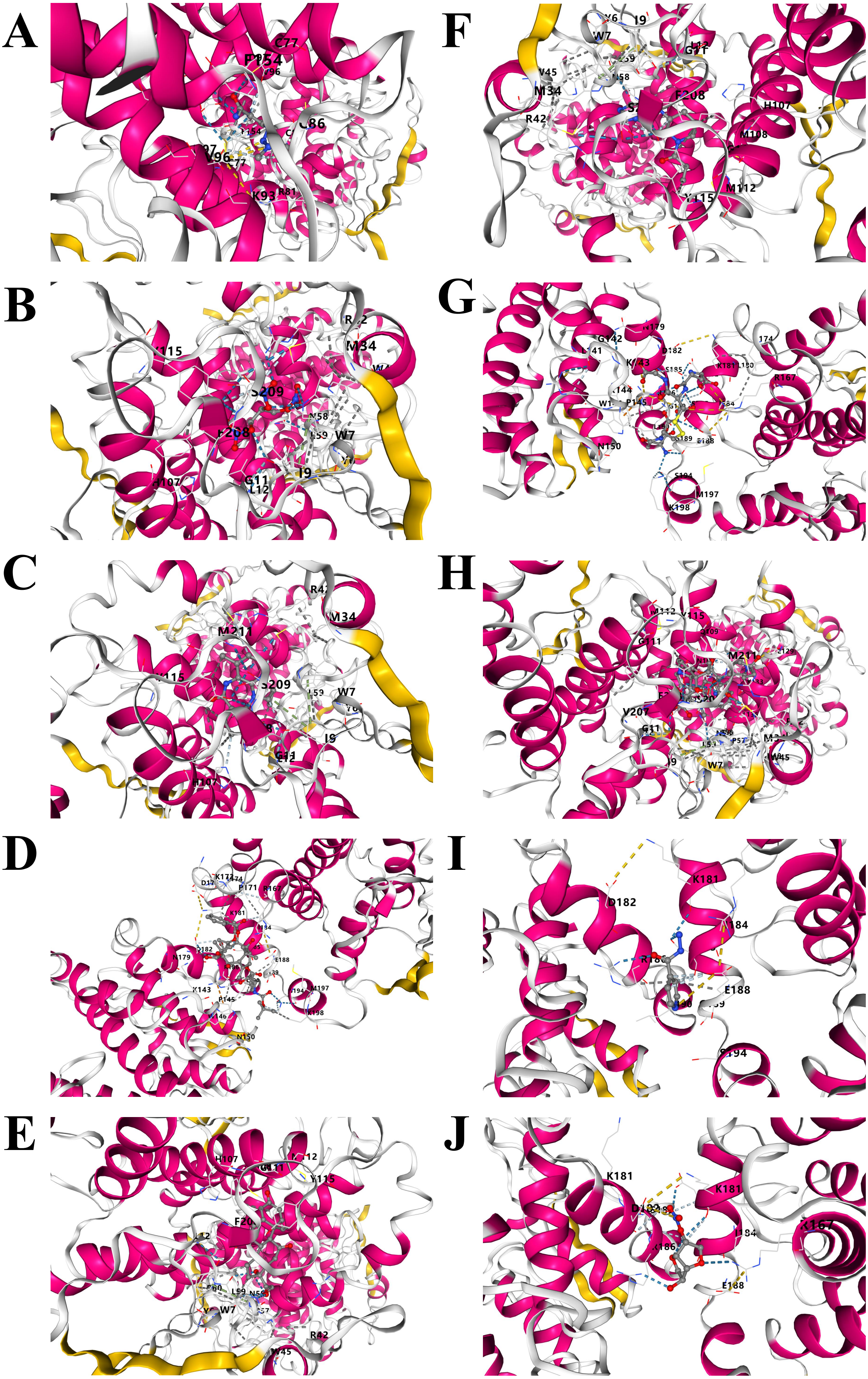
A
B
C
D
E
F
G
H
I
J

Supplementary Figure 7. Molecular docking interactions between GSTM1 and Candidate drugs ranked by binding energy.

Docking results are shown for:
(A) Dacarbazine;
(B) Nevirapine;
(C) Nitroglycerin;
(D) Docetaxel;
(E) Prednisone;
(F) Ritlecitinib;
(G) Glutathione disulfide;
(H) Vinblastine;
(I) Isoniazid;
(J) GIsosorbide mononitrate.
